# Construction and validity of an instrument for assessing the competencies of resident nurses in neonatology

**DOI:** 10.1590/0034-7167-2024-0538

**Published:** 2025-12-08

**Authors:** Mayara Mota Araujo, Alexandre Pazetto Balsanelli, Flávia Simphronio Balbino, Ana Paula Dias França Guareschi

**Affiliations:** I Universidade Federal de São Paulo. São Paulo, São Paulo, Brazil

**Keywords:** Nursing Education, Competency-Based Education, Neonatal Nursing, Educational Assessment, Validation Study., Educación en Enfermería, Educación Basada en Competencias, Enfermería Neonatal, Evaluación Educacional, Estudio de Validación.

## Abstract

**Objectives:**

to construct and validate the content of an instrument for assessing the competencies of resident nurses in neonatology.

**Methods:**

this descriptive study involved the construction and validity of a measuring instrument. Stage 1 involved a literature review to establish the conceptual framework, define competencies and objectives, and select and organize items. Stage 2 involved developing the instrument. Stage 3 involved seven expert judges who validated the content using the Delphi technique, with a Content Validity Index greater than or equal to 80%.

**Results:**

the score given by expert judges, in the first round, ranged from 0.572 to 1.00, with an average of 0.901 and a standard deviation of 0.161, and in the second round, it showed a value of 1.00.

**Conclusions:**

the instrument for assessing the competencies of resident nurses in neonatology has had its content validated, and its psychometric properties can now be tested.

## RESUMEN

**Objetivos:**

construir y validar el contenido del instrumento de competencias para enfermeras residentes en neonatología.

**Métodos:**

investigación descriptiva sobre la construcción y validación de un instrumento de medición. En la etapa 1, se realizó una revisión bibliográfica para establecer el marco conceptual, definir las competencias y los objetivos, y seleccionar y organizar los ítems. En la etapa 2, se desarrolló el instrumento. En la etapa 3, participaron siete jueces expertos, quienes validaron el contenido mediante la técnica Delphi con un Índice de Validez de Contenido igual o superior al 80%.

**Resultados:**

la puntuación otorgada por los jueces expertos en la primera ronda osciló entre 0,572 y 1,00, con una media de 0,901 y desviación estándar de 0,161, y en la segunda ronda, arrojó un valor de 1,00.

**Conclusiones:**

el instrumento de competencias para enfermeras residentes en neonatología ha sido validado en su contenido, y sus propiedades psicométricas pueden ser probadas.

## INTRODUCTION

Nursing in neonatal health ensures the adequate newborn growth and development, with a reduction in neonatal morbidity and mortality, through effective and safe care^(1)^. In this regard, there is a need to create an organizational environment where the work process is structured with the articulation of care, management, educational, investigative, and political aspects^(2)^, according to newborns’ and their families’ needs. Nurses are the professionals with the necessary skills to perform and manage nursing care with resolution^(3)^.

The training of generalist nurses involves acquiring skills that guide their performance, such as management, educational, leadership, decision-making, and communication skills^(4)^. These skills are refined during specialization in neonatal nursing, when other skills are developed, such as management of human, physical, and material resources; care management; construction of indicators; patient safety; and development of procedure protocols^(5)^.

From the theoretical basis that shows the concept of competencies, it is understood that competency is the ability to act effectively in a certain type of situation supported by knowledge and is also based on intuitive processes and resolution processes, enabling the development of action strategies^(6)^. In other words, competency is a responsible and recognized knowing how to act that implies mobilizing, integrating, transferring knowledge, resources and skills that add economic value to the organization and social value to individuals^(7)^.

Recognizing the impact of neonatal nursing care on neonatal health indicators, the Ministries of Education and Health have invested in promoting neonatal nursing residency programs. These programs are designed to qualify nurses as experts, equipping them with a humanitarian and ethical profile and the skills necessary to strengthen the neonatal population’s health^(8)^.

In order to acquire the necessary skills to become a specialist in neonatal nursing, the Pedagogical Policy Projects of residency programs incorporate resident monitoring and development strategies, including the definition of learning goals^(9)^.

Within a specialization program, such as a professional nursing residency program in neonatal health, a teaching approach is adopted that focuses on resident nurses’ competencies. The goal is to ensure that future expert nurses possess the knowledge, skills, desired attitudes, and values through a methodology of problem-solving and meaningful learning^(10)^.

The implementation of assessment metrics in residents’ teaching-learning process plays a fundamental role in monitoring their skills acquisition. Although the literature presents a variety of instruments aimed at guiding nursing care, there is a notable lack of research related to neonatal nursing experts’ specific skills.

Given the above, the question is: does the instrument for assessing the competencies of resident nurses in neonatology cover the competencies required for this expert during their training? Based on this reflection, this research aimed to develop and validate the content of an instrument developed to assess the competency of resident nurses in neonatology.

## OBJECTIVES

To construct and validate the content of an instrument for assessing the competencies of resident nurses in neonatology.

## METHODS

### Ethical aspects

The study was conducted in accordance with national and international ethics guidelines and approved by the *Universidade Federal de São Paulo* Research Ethics Committee.

### Design, period and location

This is descriptive research focusing on content development and validity of a measurement instrument, the results of which were organized based on the Equator Guidelines for Reporting Reliability and Agreement Studies precepts^(11)^. However, due to the peculiarities of the instrument analysis process, adaptations were made.

The study was developed in three stages: (1) instrument item structuring (in 2019); (2) content validity (in 2022); and (3) final composition of the instrument (in 2023).

### Step 1 - Instrument item structuring

To establish the conceptual framework, define competencies and objectives, assess the response scale, and select and organize items, a narrative review was conducted. Articles were searched in the US National Library of Medicine, National Institutes of Health, Medical Literature Analysis and Retrieval System Online, Latin American and Caribbean Literature in Health Sciences, and the Scientific Electronic Library Online and Australian College of Nursing databases. The search strategies used the Health Sciences Descriptors for the terms “nursing education”, “competency-based education”, “professional competency”, “neonatal nursing”, and “educational assessment” in English, Spanish, and Portuguese, with the Boolean operator AND adapted to each database.

Based on the literature review, the prototype of an instrument named “*Instrumento de avaliação das competências do residente de enfermagem em neonatologia*” (ICEN) was developed and structured. This instrument was formulated by the Nursing Residency Program in Neonatology at a federal university in the state of São Paulo, based on Fleury; Fleury^(6)^, Perrenoud^(7)^ theoretical framework and the Brazilian Society of Pediatric Nurses, undone by Gaiva MAM *et al*.^(12)^. The identified requirements were grouped into large domains, which are characterized by competencies, in which each competency defines specific objectives that, consequently, allow the construction of expert residents’ activities and attitudes.

### Step 2 - Content validity

Expert judges were selected. The sample size was determined by convenience, following Pasquali’s recommendations^(13)^. In this study, seven expert judges participated in the competency assessment instrument content validity, meeting the following inclusion criteria: being an expert nurse, coordinator, and/or tutor of neonatal nursing residency programs; and having experience in nursing care for newborns and their families. Program coordinators and tutors from other healthcare professional fields were excluded. Expert search was conducted through CV assessment on the Brazilian National Council for Scientific and Technological Development *Lattes* Platform (http://lattes.cnpq.br/), through the subject criterion (neonatal nursing residency) and filters (academic training/qualification, professional activity, and institution), publications in journals and/or by recommendation of experts.

Experts were emailed invitation letters, which outlined the study objectives and described their participation. They completed a characterization questionnaire and an online form containing the proposed items for ICEN content validity. Upon acceptance, each judge received ICEN and a link to Google Docs via email for analysis.

The data collection instrument was a questionnaire composed of two parts: A and B. Part A contains seven multiple-choice questions about staff characteristics, including sex, age, qualifications, length of training and experience in the neonatal unit and/or residency program, and their role. Meanwhile, Part B contains 16 questions on a Likert-type scale: five questions addressing “assessment of competencies of resident nurses in neonatology regarding their appearance and clarity”, ten questions addressing “assessment of competencies of resident nurses in neonatology regarding their comprehensiveness and relevance to the practice of nurses working in the context of newborn and family healthcare”, and one question addressing “assessment of competencies of resident nurses in neonatology regarding their applicability to the practice of neonatal nursing residency programs”.

The panel of judges validated and determined the instrument’s reliability through a four-point Likert-based assessment questionnaire: 1 = strongly agree, understanding that an item is relevant and does not need revision; 2 = agree, understanding that an item is relevant but requires minor revision; 3 = disagree, understanding that an item is relevant but requires major revision; 4 = strongly disagree, understanding that an item is not relevant. Judges were asked to analyze ICEN content to definitively include or exclude items, and a space was left in the evaluators’ questionnaire for suggestions for improving the items or commenting on the subject being assessed.

### Step 3 - Final composition of the instrument

Changes were made to the items according to judges’ considerations, and the updated version of ICEN was presented, consisting of competencies, specific objectives, and activities/attitudes expected of resident nurses in neonatology.

### Analysis of results and statistics

To verify and assess ICEN’s psychometric properties, the Content Validity Index (CVI) was applied. A CVI was calculated for each item based on Likert scale scores 1 and 2, which refer to the agreement among judges for a given item. Thus, to calculate CVI for each item, the following formula was defined: number of responses that obtained scores of 1 and 2 divided by the total number of responses for the same item. Agreement of at least 80% among judges served as the criterion for deciding an item’s relevance and acceptance^(13)^, i.e., the acceptable CVI agreement index was at least 0.80. In cases where this value was not reached, another round of assessment for the item was performed. The calculation was based on the variance of individual items and the variance of the sum of items for each judge, for all items in the questionnaire, using the same measurement scale^(14)^.

## RESULTS

ICEN was structured with five competencies defined in evidence-based clinical practices, continuing education, communication and interpersonal and interprofessional relationships, leadership, administration and management, 26 specific objectives, and 168 fundamental activities/attitudes (Chart 1) for building the learning of resident nurses in neonatology, assigned by the degree of complexity of care for newborns and their families during the residents’ 24-month training period.

**Chart 1:** The five competencies, 26 objectives, and fundamental activities/attitudes for building the learning of resident nurses in neonatology from the “*Instrumento de avaliação das competências do residente de enfermagem em neonatologia*”, São Paulo, São Paulo, Brazil, 2023

COMPETENCIES	SPECIFIC OBJECTIVES	ACTIVITIES DEVELOPED
**Evidence-based clinical practice**	Implement public policies for prenatal, labor, birth, newborn, and family care, utilizing referral and counter-referral services.	Family guidance on government programs related to the perinatal period
	Analyze newborns’ health-disease process, focusing on safe education, protection, recovery, and health promotion actions, as well as prevention of harm to newborns and families.	Guidance on the importance of immunization and the first vaccines
	Implement educational programs aimed at preventing neonatal risks and improving health indicators in all care settings.	Family guidance on infection prevention
	Implement Childand Family-Centered Care.	Fostering family participation in newborn care
	Utilize material resources and equipment for neonatal care.	Knowledge of materials and techniques for collecting samples
	Apply knowledge and skills in semiology and semiotics to the neonatal nursing work process.	Performing a physical examination of the newborn and correlating the findings with pathophysiology and clinical history
	Develop decision-making skills in neonatal nursing care.	Autonomy and confidence in care decisions
	Evidence-based clinical practice.	Sharing decision-making with the multidisciplinary team and the family
	Develop the Nursing Process for newborns, identifying risks and clinical complications.	Development of the stages of the Nursing Process based on clinical reasoning
**Continuing education/in-service education**	Conduct case studies focused on comprehensive care for newborns and their families.	Presentation of case studies at clinical meetings
	Show an interest in the pursuit of knowledge as a fundamental element of clinical practice.	Preparation of the weekly field report
	Conduct critical readings on public policies, standards, and the functioning of the neonatal unit, pathophysiological processes, and nursing care for newborns.	Institutional protocols
	Identify the team’s continuing education needs and plan educational projects to improve neonatal practice.	Identification of the educational action priority with the neonatal unit manager
	Conduct research that addresses improvements in the neonatal practice environment.	Developing and/or updating institutional protocols
	Participate in the assessment processes for nursing team members.	Participating alongside nurses and managers in the neonatal unit nursing staff assessment
**Communication and interpersonal and interprofessional relationships**	Demonstrate verbal and nonverbal communication appropriate for the neonatal unit environment.	Nonverbal communication compatible with the nurse’s profile regarding posture, touch, use of space, and gestures
	Perform clear, objective written communication in accordance with Brazilian Portuguese norms and organizational culture.	Complete nursing records, using Portuguese language standards, with clarity and objectivity
	Demonstrate dignity and respect toward the family.	Welcoming and therapeutic listening to the newborn’s family
	Develop assertive relationships within the interprofessional team.	Implementing strategies for conflict prevention and mediation
	Use Information and Communication Technologies in neonatal care.	Using technological resources in the teaching-learning process and neonatal care
**Leadership**	Act interdisciplinarily in planning care for newborns and their families.	Discussion of clinical cases and procedures with the multidisciplinary team
	Develop a professional profile that encompasses reflective, critical, humanitarian, political, and ethical attitudes, with responsibility and competency for neonatal nursing.	Knowledge of the legislation that supports the nurses’ role in caring for newborns and their families
	Critically analyze their role as a professional citizen in changing the Brazilian reality of neonatal care.	Presentation of justifications for their actions, in addition to direct nursing care, with reflection on transforming reality
	Identify the leadership profiles of neonatal unit nurses and correlate them with theoretical models.	Identification of the profile of neonatal unit nurses according to theoretical models
	Develop leadership characteristics for neonatal unit nurses.	Lead by example
**Administration and management**	Participate in the neonatal unit administration and management.	Participation in the admission of medium and high-risk newborns to the neonatal unit

*Note: although this instrument was developed and validated in Brazilian Portuguese, this chart was translated to improve understanding for foreign readers.*

The “evidence-based clinical practice” competency consisted of nurses’ exclusive, systematic care, demonstrating critical thinking and clinical reasoning for decision-making. It led to the development of the following specific objectives: implement public policies for pre-delivery, labor, and birth care, as well as for newborns and family; analyze newborns’ health-disease process; implement educational programs aimed at preventing neonatal risks and improving health indicators; provide newbornand family-centered care; utilize neonatal care material resources and equipment; apply knowledge and skills in semiology and semiotics; develop decision-making skills; and develop the Systematization of Nursing Care.

The “continuing education” competency was based on residents’ constant pursuit of learning and professional qualification, enabling the development of specific objectives such as: demonstrate an interest in the pursuit of knowledge; conduct critical readings on public policies, standards and service functioning, pathophysiological processes and nursing care; consume research that contemplates the improvement of neonatal practice environments; diagnose the team’s continuing education demands and planning educational projects; and participate in the assessment processes of nursing staff members.

The “communication and interpersonal and interprofessional relationships” competency was developed based on exchange of information, communication, and interaction between professionals and the work environment, which determined the following objectives: present adequate verbal and nonverbal communication; carry out clear and objective written communication; demonstrate dignity and respect towards the family; develop an assertive relationship with the interprofessional team; and use Information and Communication Technologies in neonatal care.

The “leadership” competency defined the following specific objectives: act interdisciplinarily in care planning; develop a professional profile that encompasses reflective, critical, humanitarian, political, and ethical attitudes with responsibility and competency; critically analyze their role as a professional citizen in changing the Brazilian reality of neonatal care; identify the leadership profiles of nurses in neonatal care services and relate them to theoretical models; and develop leadership characteristics of nurses in neonatal care services.

The “administration and management” competency led to a specific objective, which was to participate in healthcare service administration and management.

Seven expert judges participated in the study, all female, ranging in age from 36 to ≥51 years. All judges were expert nurses with 16 to 20 years of experience since graduation. The highest degree among judges was one judge with a postdoctoral degree (14.3%), five with a doctoral degree (71.4%), and one judge with a master’s degree (14.3%). According to the qualifications presented, three of the judges are experts in pediatrics and neonatology/pediatrics, and childcare (42.9%); three have a degree only in neonatology (28.6%); one judge has a specialization in public health (14.3%); and one judge has a specialization in epidemiology (14.3%).

In relation to expert nurses’ professional performance, two are coordinators of neonatology nursing residency programs (28.6%); two are preceptors of neonatology nursing residency programs (28.6%); one is a tutor of a neonatology nursing residency program (14.3%); and two work as nurses in a neonatal unit (28.6%). Within the context of neonatology nursing, five of the judges work in hospital settings (71.4%), and two work in teaching (28.6%). Overall, expert nurses showed that their time working in the neonatal unit was as follows: three judges have worked for more than 21 years (42.9%); one judge has worked for between six and ten years (14.3%); and one judge has worked for five years (42.9%).

CVI calculation formula was used to assess CVI of each item, determined by inter-judge agreement on a Likert scale. Regarding the assessment of the five ICEN competencies, in the first round, conducted by expert judges, changes were suggested to ICEN content items (Table 1). Based on judges’ suggestions for the items, a new version of the instrument was developed, modifying and maintaining some items.

**Table 1:** Content Validity Index and items of the “*Instrumento de avaliação das competências do residente de enfermagem em neonatologia*”, with suggestions based on judges’ assessment in the first round, São Paulo, São Paulo, Brazil, 2023

Itens de avaliação do ICEN	IVC	Itens com sugestão dos juízes	Ajustes dos itens
**1. Appearance and clarity**			
1.1 The title reflects the document’s objectives.	1.00	Yes	Modified
1.2 The document’s language is clear and easy to understand.	1.00	Yes	Modified
1.3 The font size and typeface are appropriate for reading.	0.857	Yes	Modified
1.4 The visual composition is attractive and organized.	0.857	Yes	Modified
1.5 The competencies are presented in a logical order.	1.00	Yes	Modified
**2. Comprehensiveness and relevance**			
2.1 The objectives are consistent with the proposed activities and attitudes.	1.00	Yes	Modified
2.2 The document addresses the main public policies and guiding documents that support neonatology nurses’ competencies.	0.858	No	Maintained
2.3 The document addresses the main contexts of care provided by neonatology nurses.	1.00	No	Maintained
2.4 The division of the competency acquisition periods for resident nurses is appropriate.	0.857	No	Maintained
2.5 The five competencies address the main aspects of neonatology nurses’ practice, focused on newborn and family-centered care.	1.00	Yes	Modified
2.6 In the “evidence-based clinical practice” competency, the activities developed are organized according to specific objectives and are important for residents’ learning.	0.858	Yes	Modified
2.7 In the “continuing education” competency, the activities developed are organized according to specific objectives and are important for residents’ learning.	0.572	Yes	Modified
2.8 In the “communication and interpersonal and interprofessional relationships” competency, the activities developed are organized according to specific objectives and are important for residents’ learning.	0.857	Yes	Modified
2.9 In the “leadership” competency, the activities developed are organized according to specific objectives and are important for residents’ learning.	1.00	Yes	Modified
2.10 In the “administration and management” competency, the activities developed are organized according to specific objectives and are important for residents’ learning.	0.715	Yes	Modified
**3. Applicability**			
3.1 The skills are applicable in their work context.	1.00	No	Maintained

According to the statistical data in the first round of ICEN assessment, CVI varied from 0.572 to 1.00, with an average of 0.901 and a standard deviation of 0.161.

In the first round, judges assessed ICEN competencies in three sections: appearance and clarity, comprehensiveness and relevance, and applicability, totaling 16 items. When analyzing the content validity of each item in the assessment of competencies of resident nurses in neonatology regarding appearance and clarity, in which judges assessed characteristics such as instrument’s language, structure, and presentation, a satisfactory CVI (CVI) between 0.857 and 1.00 was notable. However, all items assessed in this section received suggestions and were modified according to expert judges’ comments.

It was observed that the items regarding the instrument’s appearance and clarity, and applicability showed satisfactory results regarding content validity, in which CVI was from 0.857 to 1.00.

However, when analyzing the content validity of each item composed in the assessment of competencies regarding their comprehensiveness and relevance for nursing practice, CVI resulted between 0.572 and 1.00, in which seven items were changed according to judges’ suggestions.

In this part of assessment, two items obtained a CVI below 0.800, with unsatisfactory validity indices: item 2.7, with a CVI of 0.572, which involves the “continuing education” competency, and item 2.10, with a CVI of 0.715, which characterizes the “administration and management” competency. These two items were modified based on judges’ opinions and, shortly thereafter, conducted for the second round of validity. The second round occurred with the instrument adjusted only in subitems 2.7 and 2.10, based on the suggestions presented in the first round.

In the first round, judges’ assessments of items 2.7 and 2.10 reinforced the study’s focus on validating and restructuring the instrument. In the “continuing education” competency, in-service education was included, heightening the need to integrate these topics into resident nurses’ learning process. The activities and attitudes for these competencies were summarized from 27 to 21 actions expected of residents. Meanwhile, in the “administration and management” competency, the activities and attitudes were reformulated to include the management of quality indicators and adjust the reading and reflection activity, potentially proposing changes to the Standard Operating Procedures.

In the second round, after adjustments, expert judges did not describe possible suggestions, and CVI was consolidated at 1.00 between the assessed subitems 2.7 and 2.10.

When verifying the content validity regarding the applicability of the instrument in nursing residency programs in neonatology, CVI set it at 1.00, and judges did not present suggestions for modification.

The final ICEN configuration, after changes in accordance with expert judges’ assessments, has five competencies, with the new competency suggested by judges, “in-service education”, included alongside the “continuing education” competency. The number of specific objectives remained unchanged, remaining at 26. Meanwhile, the activities and attitudes expected of resident nurses in neonatology underwent changes, reducing the number from 174 to 168. The expected activities and attitudes, which were modified according to judges’ assessments, were included in two competencies: “evidence-based clinical practice” and “continuing education/in-service education”.

## DISCUSSION

The literature review established the expected concepts of nursing competencies, guiding the establishment of the conceptual framework presented in ICEN. This allowed for the definition of the instrument’s competencies and objectives and, consequently, the organization of the presented items, referred to as expected attitudes. In other words, for each competency, objectives related to that competency were formulated, and for each objective, attitudes that residents need to acquire were demonstrated.

When considering neonatology nurses’ competencies, the importance of the training process is clear, as both practical and theoretical content must be directed toward preparing expert nurses for the job market. Activities that contribute to nurses’ practice within the Neonatal Intensive Care Unit include: understanding the institutional mission and values; participating in the multidisciplinary team physical structure and composition; forecasting services; managing costs; establishing patient and family care processes; developing indicators; and creating care protocols^(5)^.

Communication, decision-making, and difficulties in interpersonal relationships are challenges encountered, and nurses use management tools to resolve these conflicts, which will facilitate the process of interpersonal relationships, the establishment of bonds and the organization of the work environment^(15)^.

The “communication and interpersonal and interprofessional relationships”, “leadership” and “administration and management” competencies assessed in ICEN can help the resident in resolving related demands, as each objective contained in the instrument influences the execution of activities and attitudes that can lead residents to adequate verbal and non-verbal communication for an assertive relationship that allows critical and reflective attitudes, in addition to expanding the managerial performance of the resources available to provide qualified and safe nursing care^(16)^.

Another study found that neonatology nurses’ competencies are organized into domains that include ethical and legal practice, clinical practice, management, leadership, teamwork, research, knowledge production, and educational practice^(12)^. The data from this study identify competencies, prompting the introduction of methodologies that promote relevant learning around these competencies throughout specialization. Therefore, one way to ensure these competencies are developed during specialization is to introduce an assessment tool like ICEN. Through comparative analysis, the domains presented in this study are consistent with competencies established and developed in ICEN itself, which also considers residents’ competency level gradually.

It is essential to understand the instruments and methods applicable to assessing professional competency in nursing. Assessment methodologies imply maintaining teaching-learning actions and strategies and modifying professional qualification proposals^(17)^.

Recently, nursing education has embraced competency-based education. A competency-based curriculum provides an understanding of knowledge, skills, and experiences, with students being critically assessed on assignments, enhancing critical thinking and providing training opportunities^(18,19)^.

Another point is based on the General Guidelines for Multiprofessional Residency Programs and Healthcare Professionals, specifying that the pedagogical project of a residency program must provide methodologies for integrating knowledge and practices that allow for the construction of shared skills, considering the need for changes in the processes of training, care and management in health^(20)^.

It was observed that, during judges’ assessment of ICEN items, some modifications were made to the items, while other items remained unchanged. This occurred because experts reflected on the appropriate assessment of nurses and on what the assessment instrument should be like to enable residency training learning and monitoring so that it meets professionals’ theoretical and practical frameworks. Modifications were created from a single round of assessment among judges, as this round allowed for the alignment of the content with the proposed objective.

Thus, the study’s assessment instrument is well-founded and valid based on its content. Its structure is clear and comprehensive, and its relevant content is comprehensive and relevant. This content should be assessed during residency training, while maintaining its applicability across neonatal nursing residency programs. The objective is to assist coordinators, tutors, and preceptors in assessing residents during practice settings, allowing for testing its psychometric properties.

### Study limitations

A limitation of this study is the lack of instruments with the same scope that would allow for comparison of results or the conduct of a comparative study. Another limitation is that ICEN requires the assessment of nursing residents’ competencies, restricting its application to uniprofessional nursing residency programs in neonatology.

### Contribution to nursing and health

The study presents implications for future research, as it addresses topics related to the care of neonatology nurses, management and administration in neonatology, and teaching in specialization, with a focus on nursing residency in neonatology.

## CONCLUSIONS

This study validated ICEN content based on analysis and suggestions from expert judges. Thus, the final configuration of the instrument has 26 specific objectives and 168 activities and attitudes fundamental to building the learning of resident nurse in neonatology over a 24-month period.

ICEN was considered a valid and reliable alternative for assessing expert nurses. It is expected that ICEN can be applied to neonatal nursing residents in residency programs, facilitating competency assessment, fostering nursing education, and assisting residents in their own development, enabling them to build their own reflection and critical analysis of the knowledge imparted.

## Data Availability

The research data are available in a repository: https://doi.org/10.48331/scielodata.6MRF78.
