# Supplementary material for: Construction and validity of an instrument for assessing the competencies of resident nurses in neonatology
Source: Rev Bras Enferm. 2025 Dec 8;78(Suppl 3):e20240538. doi: 10.1590/0034-7167-2024-0538 (PMC12707849; doi:10.1590/0034-7167-2024-0538)
Supplement: Translation

# Translation: Construção e validação de instrumento de avaliação das competências do enfermeiro residente em neonatologia

**DOI:** 10.1590/0034-7167-2024-0538pt

**Authors:** Mayara Mota Araujo, Alexandre Pazetto Balsanelli, Flávia Simphronio Balbino, Ana Paula Dias França Guareschi

**Affiliations:** I Universidade Federal de São Paulo. São Paulo, São Paulo, Brasil

**Keywords:** Educação em Enfermagem, Educação Baseada em Competências, Enfermagem Neonatal, Avaliação Educacional, Estudo de Validação.

## Abstract

**Objetivos:**

construir e validar o conteúdo de instrumento de competências do enfermeiro residente em neonatologia.

**Métodos:**

pesquisa descritiva de construção e validação de instrumento de medida. Na etapa 1, ocorreram a revisão de literatura para o estabelecimento da estrutura conceitual, definição das competências e objetivos, seleção, e organização dos itens. Na etapa 2, ocorreu a elaboração do instrumento. Na etapa 3, participaram sete juízes especialistas, que validaram o conteúdo através da técnica Delphi com Índice de Validade de Conteúdo maior ou igual a 80%.

**Resultados:**

a pontuação feita pelos juízes especialistas, na primeira rodada, variou de 0,572 a 1,00, com média de 0,901 e desvio padrão de 0,161, e na segunda rodada, mostrou o valor de 1,00.

**Conclusões:**

o instrumento de avaliação de competências do enfermeiro residente em neonatologia teve seu conteúdo validado, e agora pode ter suas propriedades psicométricas testadas.

## INTRODUÇÃO

A enfermagem na saúde neonatal garante o crescimento e o desenvolvimento adequado do recém-nascido, com redução da morbimortalidade neonatal, por meio de uma assistência efetiva e segura^(1)^. Nesse sentido, há necessidade de criar um ambiente organizacional onde o processo de trabalho seja estruturado com articulação dos aspectos assistenciais, gerenciais, educativos, investigativos e políticos^(2)^, conforme as necessidades do recém-nascido e sua família. O enfermeiro é o profissional com as competências necessárias para executar e gerenciar a assistência de enfermagem com resolutividade^(3)^.

Na formação do enfermeiro generalista, é prevista a aquisição de competências que balizam a sua atuação, como as competências gerenciais, educacionais, liderança, tomada de decisão e comunicação^(4)^, que são lapidadas, posteriormente, durante a especialização da enfermagem neonatal, processo em que há a ampliação de outras competências, como a gestão de recursos humanos, físicos e de materiais, gestão do cuidado, construção de indicadores, segurança do paciente, e elaboração de protocolos de procedimentos^(5)^.

Tendo como ponto de vista a base teórica que mostra o conceito de competências, compreende-se que competência é a capacidade de atuar eficazmente em determinado tipo de situação amparada em conhecimento e também se baseia em processos intuitivos e processos de resolução, possibilitando o desenvolvimento de estratégias de ação^(6)^. Ou seja, a competência é um saber agir responsável e reconhecido que implica mobilizar, integrar, transferir conhecimentos, recursos e habilidades que agreguem valor econômico à organização e valor social ao indivíduo^(7)^.

Ao reconhecer o impacto da assistência de enfermagem neonatal nos indicadores de saúde neonatal, os Ministérios da Educação e Saúde têm investido na promoção de programas de residência em enfermagem neonatal. Esses programas são projetados para qualificar enfermeiros como especialistas, equipando-os com um perfil humanitário e ético, e com competências necessárias para fortalecer a saúde da população neonatal^(8)^.

No sentido de adquirir as competências necessárias para se tornar um especialista em enfermagem neonatal, os Projetos Políticos Pedagógicos dos programas de residência incorporam estratégias de acompanhamento e desenvolvimento do residente, incluindo a definição de metas de aprendizagem^(9)^.

Dentro de uma especialização, como um programa de residência profissional em saúde de enfermagem em neonatologia, é adotada uma abordagem de ensino que se concentra nas competências do enfermeiro residente. O objetivo é assegurar que o futuro enfermeiro especialista tenha conhecimentos, habilidades, atitudes desejadas e valores por meio da metodologia de problematização e aprendizagem significativa^(10)^.

A implementação de métricas avaliativas no processo de ensino-aprendizagem do residente desempenha um papel fundamental no acompanhamento na sua aquisição de competências. Embora a literatura apresente uma variedade de instrumentos voltados à orientação da assistência de enfermagem, há uma notável carência de pesquisas relacionadas às competências específicas do especialista em enfermagem neonatal.

Diante do exposto, questiona-se: o instrumento de avaliação de competências do residente de enfermagem neonatal abrange as competências necessárias para este especialista durante a sua formação? E a partir dessa reflexão, o objetivo da investigação foi construir e validar o conteúdo do instrumento elaborado para avaliação de competência do enfermeiro residente especialista em neonatologia.

## OBJETIVOS

Construir e validar o conteúdo de instrumento de competências do enfermeiro residente em neonatologia.

## MÉTODOS

### Aspectos éticos

O estudo foi conduzido de acordo com as diretrizes de ética nacionais e internacionais, e aprovado pelo Comitê de Ética em Pesquisa da Universidade Federal de São Paulo.

### Desenho, período e local

Trata-se de pesquisa descritiva com foco na elaboração e validação do conteúdo de um instrumento de medida, cujo resultados foram organizados com base nos preceitos da ferramenta *Guidelines for Reporting Reliability and Agreement Studies* do Equator^(11)^. Porém, devido às peculiaridades do processo de análise do instrumento, foram realizadas adaptações.

O estudo foi desenvolvido em três etapas: (1) estruturação dos itens do instrumento (em 2019); (2) validação do conteúdo (em 2022); e (3) composição final do instrumento (em 2023).

### Etapa 1 - Estruturação dos itens do instrumento

Para o estabelecimento da estrutura conceitual, definição das competências e objetivos, escala de resposta, seleção e organização dos itens, foi realizada revisão narrativa com a busca de artigos nas bases de dados *US National Library of Medicine National Institutes of Health, Medical Literature Analyses and Retrieval System onLine*, Literatura Latino Americana e do Caribe em Ciências da Saúde, além de na biblioteca virtual *Scientific Electronic Library Online* e *Australian College of Nursing*. Nas estratégias de busca, foram utilizados os Descritores de Ciências da Saúde dos termos “educação em enfermagem”, “educação baseada em competências”, “competência profissional”, “enfermagem neonatal” e “avaliação educacional”, nos idiomas inglês, espanhol e português, com a combinação do operador booleano *AND* adaptado a cada uma das bases de dados.

A partir da revisão da literatura, ocorreu a elaboração e estruturação do protótipo do instrumento nomeado como “Instrumento de avaliação das competências do residente de enfermagem em neonatologia” (ICEN), formulado pelo Programa de Residência em Enfermagem em Neonatologia de uma universidade federal do estado de São Paulo, pautada no referencial teórico de Fleury; Fleury^(6)^, Perrenoud^(7)^ e Sociedade Brasileira de Enfermeiros Pediatras, desfeita por Gaiva MAM *et al*.^(12)^. Os requisitos identificados foram agrupados em grandes domínios, que se caracterizam em competências, em que cada competência define os objetivos específicos que, consequentemente, permite a construção de atividades e atitudes do residente especialista.

### Etapa 2 - Validação de conteúdo

Foi realizada a escolha dos juízes especialistas. O tamanho amostral foi definido por conveniência, respeitando as recomendações de Pasquali^(13)^. Neste estudo, sete juízes especialistas participaram da validação do conteúdo do instrumento de avaliação de competências, atendendo aos seguintes critérios de inclusão: ser enfermeiro especialista, coordenador e/ou tutor de programas de residência em enfermagem neonatal; e possuir atuação na assistência de enfermagem ao recém-nascido e sua família. Foram excluídos os coordenadores e tutores de programa que são de outras áreas profissionais de saúde. A busca dos especialistas ocorreu através da avaliação dos currículos na Plataforma *Lattes* (http://lattes.cnpq.br/) do Conselho Nacional de Desenvolvimento Científico e Tecnológico, por meio do critério assunto (residência enfermagem neonatal) e filtros (formação acadêmica/titulação, atividade profissional e instituição), publicações em periódicos e/ou por indicação de especialistas.

Aos especialistas, foram enviadas cartas convite por e-mail, com apresentação dos objetivos do estudo e a descrição da participação, através de preenchimento de questionário de caracterização e do formulário *online*, com os itens propostos para validação de conteúdo do ICEN. Após o aceite, cada juiz recebeu por e-mail o ICEN e o *link* do formulário eletrônico do *Google Docs* para análise do instrumento.

O instrumento de coleta de dados foi um questionário composto por duas partes: A e B. A parte A possui sete questões de múltiplas escolhas sobre caracterização dos colaboradores, como sexo, idade, titulação, tempo de formação e de atuação na área na unidade neonatal e/ou programa de residência e função que exerce. Enquanto isso, a parte B possui 16 questões conforme a escala de Likert, sendo cinco questões sobre “avaliação das competências do enfermeiro residente em neonatologia quanto à aparência e clareza”, dez questões sobre “avaliação das competências do enfermeiro residente em neonatologia quanto à sua abrangência e pertinência para a prática do enfermeiro que atua nos contextos de atenção à saúde do recém-nascido e sua família” e uma questão sobre “avaliação das competências do enfermeiro residente em neonatologia quanto à aplicabilidade na prática dos programas de residência de enfermagem neonatal”.

O grupo de juízes validou e determinou a confiabilidade do instrumento através do questionário de avaliação baseado em escala de Likert de 4 pontos: 1 = concordo fortemente, entendendo que o item é relevante e não precisa de revisão; 2 = concordo, entendendo que o item é relevante, porém necessita de pequena revisão; 3 = discordo, entendendo que o item é relevante, porém necessita de grande revisão; 4 = discordo fortemente, entendendo que o item não é relevante. Aos juízes, foi solicitada a análise do conteúdo do ICEN para inclusão ou exclusão definitiva de itens, e foi deixado um espaço, no questionário desses avaliadores, para que eles escrevessem sugestões para melhorar os itens ou fazer comentários sobre o objeto avaliado.

### Etapa 3 - Composição final do instrumento

Foram realizadas alterações dos itens de acordo com as considerações dos juízes, e a versão atualizada do ICEN foi apresentada, sendo composta por competências, objetivos específicos e atividades/atitudes esperadas do enfermeiro residente em neonatologia.

### Análise dos resultados e estatística

Para verificação e avaliação das propriedades psicométricas do ICEN, aplicou-se o Índice de Validade de Conteúdo (IVC). Foi calculado um IVC para cada item de acordo com as pontuações 1 e 2 da escala Likert, que se referem à concordância entre os juízes para determinado item avaliado. Dessa maneira, para o cálculo do IVC de cada item, foi definida a seguinte fórmula: nº de respostas que obtiveram pontuação 1 e 2 dividido pelo nº total de respostas do mesmo item. A concordância de pelo menos 80% entre os juízes serviu de critério de decisão sobre a pertinência e aceitação do item^(13)^, ou seja, o índice de concordância aceitável do IVC foi de no mínimo 0,80. Em casos em que este valor não foi atingido, ocorreu outra rodada de avaliação para o item. O cálculo foi feito a partir da variância dos itens individuais e da variância da soma dos itens de cada juiz, de todos os itens do questionário, utilizando-se a mesma escala de medição^(14)^.

## RESULTADOS

O ICEN foi estruturado com cinco competências definidas em práticas clínica baseada em evidências, educação permanente, comunicação e relacionamento interpessoal e interprofissional, liderança, administração e gerenciamento, 26 objetivos específicos, e 168 atividades/atitudes fundamentais (Quadro 1) para construção da aprendizagem do enfermeiro residente em neonatologia, atribuídas por grau de complexidade do atendimento ao recém-nascido e sua família durante o período de formação do residente em 24 meses.

**Quadro 1:** As cinco competências, 26 objetivos e atividades/atitudes fundamentais para a construção da aprendizagem do enfermeiro residente em neonatologia do “Instrumento de avaliação das competências do residente de enfermagem em neonatologia”, São Paulo, São Paulo, Brasil, 2023

COMPETÊNCIAS	OBJETIVOS ESPECÍFICOS	ATIVIDADES DESENVOLVIDAS
**Prática Clínica Baseada em Evidências**	Aplicar as políticas públicas de atenção ao pré-natal, parto, nascimento, neonato e família, utilizando os serviços de referência e contra referência.	Orientação à família sobre os programas governamentais referente ao período perinatal
	Analisar o processo saúde-doença do recém-nascido, tendo como foco ações seguras de educação, proteção, recuperação e promoção em saúde e prevenção de agravos ao recém-nascido e à família.	Orientação sobre a importância da imunização e das primeiras vacinas
	Realizar programas educativos visando à prevenção de riscos neonatais e a melhoria dos indicadores de saúde em todos os cenários de cuidado.	Orientação à família sobre prevenção de infecção
	Realizar o Cuidado Centrado na Criança e Família.	Favorecimento da participação da família no cuidado do RN
	Utilizar os recursos materiais e equipamentos de assistência neonatal.	Conhecimento dos materiais e técnicas para coletas de exames
	Aplicar os conhecimentos e as habilidades da semiologia e semiotécnica no processo de trabalho da enfermagem neonatal.	Realização do exame físico do RN e correlacionar os achados com a fisiopatologia e a história clínica
	Desenvolver a capacidade da tomada de decisão na assistência de enfermagem neonatal.	Autonomia e segurança nas decisões assistenciais
	Prática Clínica Baseada em Evidências.	Compartilhamento das tomadas de decisão com a equipe multiprofissional e com a família
	Desenvolver o Processo de Enfermagem ao RN identificando riscos e intercorrências clínicas.	Elaboração das etapas do Processo de Enfermagem pautadas no raciocínio clínico
**Educação Permanente / Serviço**	Realizar estudos de casos pautados na atenção integral ao RN e família.	Apresentação dos estudos de casos em reuniões clínicas
	Apresentar interesse na busca do conhecimento como elemento fundamental para sua prática clínica.	Elaboração do relatório semanal de campo
	Realizar leituras críticas sobre políticas públicas, normas e funcionamento da unidade neonatal, processos fisiopatológicos e de assistência de enfermagem dos RNs.	Protocolos institucionais
	Diagnosticar as demandas de educação permanente da equipe e planejar projetos educativos para melhoria da prática neonatal.	Identificação com o gestor da unidade neonatal a prioridade de ação educativa
	Consumir pesquisas que contemplem a melhoria do ambiente de prática neonatal.	Elaboração e/ou atualização de protocolos institucionais
	Participar dos processos avaliativos dos membros da equipe de enfermagem.	Participação junto com as enfermeiras e gestoras da avaliação dos funcionários de enfermagem da unidade neonatal
**Comunicação e Relacionamento interpessoal e interprofissional**	Apresentar comunicação verbal e não verbal adequada para o ambiente da unidade neonatal.	Comunicação não verbal compatível com o perfil do enfermeiro quanto: postura, toque, utilização do espaço e gestos
	Realizar comunicação escrita clara, objetiva e de acordo com as normas da língua portuguesa e da cultura organizacional.	Registros de enfermagem completos, com uso das normas da língua portuguesa, com clareza e objetividade
	Demonstrar dignidade e respeito em relação à família.	Acolhimento e escuta terapêutica à família do RN
	Desenvolver o relacionamento assertivo na equipe interprofissional.	Implementa estratégias para prevenção e mediação de conflitos
	Utilizar as Tecnologias de Informação e Comunicação no cuidado neonatal.	Utilização dos recursos tecnológicos no processo de ensino aprendizado e assistência neonatal
**Liderança**	Atuar de maneira interdisciplinar no planejamento do cuidado ao RN e família.	Discussão dos casos clínicos e condutas com a equipe multidisciplinar
	Desenvolver um perfil profissional que contemple atitudes reflexivas, críticas, humanitárias, políticas e éticas com responsabilidade e competência para atuação na enfermagem neonatal.	Conhecimento da legislação que respalda a atuação do enfermeiro no cuidado ao RN e família
	Analisar criticamente o seu papel como cidadão profissional na mudança da realidade brasileira da assistência neonatal.	Apresentação de justificativas das suas ações, além da assistência de enfermagem direta, com reflexão para transformação da realidade
	Identificar os perfis de liderança dos enfermeiros das unidades neonatais e correlaciona-los com os modelos teóricos.	Identificação do perfil do enfermeiro da unidade neonatal, de acordo com os modelos teóricos
	Desenvolver características de liderança do enfermeiro da unidade neonatal.	Lidera pelo exemplo
**Administração e Gerenciamento**	Participar da administração e gerenciamento da unidade neonatal.	Participação da admissão do RN de médio e alto risco na unidade neonatal

A competência “prática clínica baseada em evidências” consistiu na assistência sistematizada privativa do enfermeiro com exposição do pensamento crítico e raciocínio clínico para a tomada de decisão. Conduziu a construção dos seguintes objetivos específicos: aplicar as políticas públicas de atenção ao pré-parto, parto e nascimento, neonato e família; analisar o processo de saúde-doença do recém-nascido; realizar programas educativos, visando à prevenção de riscos neonatais e melhoria dos indicadores de saúde; realizar o cuidado centrado no recém-nascido e família; utilizar os recursos materiais e equipamentos de assistência neonatal; aplicar os conhecimentos e as habilidades da semiologia e semiotécnica; desenvolver a capacidade da tomada de decisão; e desenvolver a Sistematização da Assistência de Enfermagem.

A competência “educação permanente” foi baseada na constante busca pela aprendizagem e qualificação profissional do residente, possibilitando a construção de objetivos específicos como: apresentar interesse na busca do conhecimento; realizar leituras críticas sobre políticas públicas, normas e funcionamento do serviço, processos fisiopatológicos e de assistência de enfermagem; consumir pesquisas que contemplem a melhoria do ambiente de prática neonatal; diagnosticar as demandas de educação permanente da equipe e planejar projetos educativos; e participar dos processos avaliativos dos membros de enfermagem.

A competência “comunicação e relacionamento interpessoal e interprofissional” foi desenvolvida com base na troca de informação, comunicação e interação dos profissionais com o ambiente de trabalho que determinou os seguintes objetivos: apresentar comunicação verbal e não verbal adequada; realizar comunicação escrita, clara e objetiva; demonstrar dignidade e respeito em relação à família; desenvolver o relacionamento assertivo com a equipe interprofissional; e utilizar as Tecnologias da Informação e Comunicação no cuidado neonatal.

A competência “liderança” definiu os seguintes objetivos específicos: atuar de maneira interdisciplinar no planejamento do cuidado; desenvolver um perfil profissional que contemple atitudes reflexivas, críticas, humanitárias, políticas e éticas com responsabilidade e competência; analisar criticamente o seu papel como cidadão profissional na mudança da realidade brasileira da assistência neonatal; identificar os perfis de liderança dos enfermeiros nos serviços de atendimentos neonatais e relacioná-los com os modelos teóricos; e desenvolver características de liderança do enfermeiro nos serviços de atendimento neonatal.

A competência “administração e gerenciamento” conduziu a um objetivo específico, que foi participar da administração e gerenciamento dos serviços de saúde.

Participaram do estudo sete juízes especialista, todos do sexo feminino, cuja idade variou de 36 a ≥ 51 anos. Todos os juízes eram enfermeiros especialistas com tempo de graduação de 16 a 20 anos. A titulação máxima entre os juízes foi um juiz com pós-doutorado (14,3%), cinco com doutorado (71,4%) e um juiz com mestrado (14,3%). De acordo com a titulação apresentada, três dos juízes são especialistas em pediatria e neonatologia/pediatria, e puericultura (42,9%); três possuem titulação somente em neonatologia (28,6%); um juiz possui especialização em saúde pública (14,3%); e um juiz possui especialização em epidemiologia (14,3%).

Em relação à atuação profissional dos enfermeiros especialistas, dois são coordenadores de programas de residência em enfermagem em neonatologia (28,6%); dois são preceptores de programa de residência em enfermagem em neonatologia (28,6%); um é tutor de programa de residência em enfermagem em neonatologia (14,3%); e dois atuam como enfermeiros em unidade neonatal (28,6%). Dentro do contexto de atuação da enfermagem em neonatologia, cinco dos juízes atuam na área hospitalar (71,4%), e dois atuam no ensino (28,6%). Ao todo, os enfermeiros especialistas mostraram que o seu tempo de atuação na unidade neonatal foi o seguinte: três juízes atuam há mais de 21 anos (42,9%); um juiz atua entre seis e dez anos (14,3%); e um juiz atua há cinco anos (42,9%).

Na avaliação do IVC de cada item determinado pela concordância entre os juízes segundo a escala Likert, foi utilizada a fórmula de cálculo do IVC. Em relação à avaliação das cinco competências do ICEN, na primeira rodada, realizada pelos juízes especialistas, houve sugestões de alterações nos itens de conteúdo do ICEN (Tabela 1). De acordo com as sugestões apresentadas nos itens pelos juízes, foi construída uma nova versão do instrumento, com a modificação e manutenção de itens.

**Tabela 1:** Índice de Validação de Conteúdo e itens do “Instrumento de avaliação das competências do residente de enfermagem em neonatologia”, com sugestões de acordo com a avaliação dos juízes na primeira rodada, São Paulo, São Paulo, Brasil, 2023

Itens de avaliação do ICEN	IVC	Itens com sugestão dos juízes	Ajustes dos itens
**1. Aparência e clareza**			
1.1 O título reflete os objetivos do documento.	1,00	Sim	Modificado
1.2 A linguagem do documento está clara e de fácil entendimento.	1,00	Sim	Modificado
1.3 O tamanho da fonte e o tipo de letra estão adequados à leitura.	0,857	Sim	Modificado
1.4 A composição visual está atrativa e organizada.	0,857	Sim	Modificado
1.5 A ordem de apresentação das competências está disposta de forma lógica.	1,00	Sim	Modificado
**2. Abrangência e pertinência**			
2.1 Os objetivos estão de acordo com as atividades e atitudes propostas.	1,00	Sim	Modificado
2.2 As principais políticas públicas e documentos norteadores que dão suporte às competências do enfermeiro neonatologista foram contemplados no documento.	0,858	Não	Mantido
2.3 Os principais contextos de cuidado prestado pelo enfermeiro neonatologista foram contemplados no documento.	1,00	Não	Mantido
2.4 A divisão dos períodos para aquisição de competência do residente enfermeiro está adequada.	0,857	Não	Mantido
2.5 As cinco competências contemplam os principais aspectos da prática do enfermeiro neonatologista voltada ao cuidado centrado no neonato e família.	1,00	Sim	Modificado
2.6 Na competência “prática clínica baseada em evidências científicas”, as atividades desenvolvidas estão organizadas de acordo com os objetivos específicos e são importantes para a aprendizagem do residente.	0,858	Sim	Modificado
2.7 Na competência “educação permanente”, as atividades desenvolvidas estão organizadas de acordo com os objetivos específicos e são importantes para a aprendizagem do residente.	0,572	Sim	Modificado
2.8 Na competência “comunicação e relacionamento interpessoal e interprofissional”, as atividades desenvolvidas estão organizadas de acordo com os objetivos específicos e são importantes para a aprendizagem do residente.	0,857	Sim	Modificado
2.9 Na competência “liderança”, as atividades desenvolvidas estão organizadas de acordo com os objetivos específicos e são importantes para a aprendizagem do residente.	1,00	Sim	Modificado
2.10 Na competência “administração e gerenciamento”, as atividades desenvolvidas estão organizadas de acordo com os objetivos específicos e são importantes para a aprendizagem do residente.	0,715	Sim	Modificado
**3. Aplicabilidade**			
3.1 As competências são aplicáveis no seu contexto de trabalho.	1,00	Não	Mantido

Conforme os dados estatísticos na primeira rodada de avaliação do ICEN, observa-se a variação de IVC de 0,572 a 1,00, cuja média entre eles foi de 0,901 e desvio padrão de 0,161.

Na primeira rodada, os juízes avaliaram as competências contidas no ICEN em três partes quanto à aparência e clareza, abrangência e pertinência, e aplicabilidade, totalizando 16 itens de avaliação. Ao analisar a validação de conteúdo de cada item composto na avaliação das competências do enfermeiro residente em neonatologia quanto à aparência e clareza, em que os juízes avaliaram características como linguagem, estrutura e apresentação do instrumento, foi notável o IVC satisfatório entre 0,857 e 1,00. Contudo, todos os itens avaliados, dessa parte, receberam sugestões e foram modificados de acordo com os apontamentos dos juízes especialistas.

Observou-se que os itens quanto à aparência e clareza, e aplicabilidade do instrumento apontaram resultados satisfatórios referentes à validação de conteúdo, em que IVC foi de 0,857 a 1,00.

No entanto, ao analisar a validação de conteúdo de cada item composto na avaliação das competências quanto à sua abrangência e pertinência para a prática do enfermeiro, o IVC resultou entre 0,572 e 1,00, em que sete itens foram alterados de acordo com as sugestões dos juízes.

Ainda nessa parte de avaliação, dois itens obtiveram IVC abaixo de 0,800, com índices insatisfatórios para validação, que foram o item 2.7, com IVC de 0,572, o qual envolve a competência “educação permanente”, e o item 2.10, com IVC de 0,715, o qual caracteriza a competência “administração e gerência”. Esses dois itens foram modificados com base nas opiniões dos juízes e, logo depois, conduzidos para a segunda rodada de validação. A segunda rodada ocorreu com o instrumento ajustado apenas nos subitens 2.7 e 2.10, a partir das sugestões demonstradas na primeira rodada.

Na primeira rodada, as avaliações dos juízes pertinentes aos itens 2.7 e 2.10 fortaleceram a ideia do estudo em relação à validação e reestruturação do instrumento. Na competência “educação permanente”, foi inserida a educação em serviço, o que intensifica a necessidade de associação dessas temáticas na aprendizagem do enfermeiro residente, e as atividades e atitudes para essas competências foram sintetizadas de 27 para 21 ações que são esperadas pelo residente. Enquanto isso, na competência “administração e gerenciamento”, as atividades e atitudes foram reformuladas para inclusão da gestão dos indicadores de qualidade e ajuste da atividade sobre leitura e reflexão com possível proposição de mudanças nos Procedimentos Operacionais Padrão.

Na segunda rodada, depois dos ajustes, os juízes especialistas não descreveram possíveis sugestões, e o IVC se consolidou em 1,00 entre os subitens avaliados 2.7 e 2.10.

Ao verificar a validação de conteúdo quanto à aplicabilidade do instrumento nos programas de residência em enfermagem em neonatologia, o IVC dispôs em 1,00, e os juízes não apresentaram sugestões para modificação.

A configuração final do ICEN, após as alterações em conformidade com as avaliações dos juízes especialistas, possui cinco competências, sendo a nova competência sugerida pelos juízes, “educação em serviço”, incluída em conjunto com a competência “educação permanente”. A quantidade de objetivos específicos não apresentou alteração, continuando com 26 objetivos específicos. Enquanto isso, as atividades e atitudes esperadas do enfermeiro residente em neonatologia apresentaram alterações, o que reduziu a sua quantidade de 174 para 168. As atividades e atitudes esperadas, que foram modificadas de acordo com as avaliações dos juízes, estavam inseridas em duas competências: “práticas clínica baseada em evidências” e “educação permanente/educação em serviço”.

## DISCUSSÃO

A revisão de literatura firmou os conceitos esperados sobre competências do enfermeiro, direcionando o estabelecimento da estrutura conceitual apresentada no ICEN, permitindo a definição das competências e objetivos do instrumento e, consequentemente, da organização dos itens expostos, nomeadas de atitudes esperadas. Ou seja, para cada competência, formularam-se objetivos relacionados a essa competência, e para cada objetivo, foram mostradas atitudes que o residente necessita adquirir.

Ao pensar nas competências de um enfermeiro neonatologista, compreende-se a importância do processo de formação, em que as práticas e os conteúdos teóricos devem ser direcionados ao compromisso em preparar o enfermeiro especialista para o mercado de trabalho. As atividades que contribuem para a prática do enfermeiro dentro da Unidade de Terapia Intensiva Neonatal são: conhecer a missão e valores institucionais; participar da estrutura física e da composição da equipe multiprofissional; prever serviços; gerenciar custos; estabelecer processos de atendimento ao paciente e família; construir indicadores; e criar protocolos assistenciais^(5)^.

A comunicação, tomada de decisão e dificuldade de relacionamento interpessoal são desafios encontrados, e o enfermeiro utiliza ferramentas gerenciais para a resolução desses conflitos, que facilitarão o processo de relacionamento interpessoal, o estabelecimento dos vínculos e a organização do ambiente de trabalho^(15)^.

As competências “comunicação e relacionamento interpessoal e interprofissional”, “liderança” e “administração e gerenciamento” avaliadas no ICEN podem auxiliar o residente na resolução das demandas relacionadas, pois cada objetivo contido no instrumento influencia a execução de atividades e atitudes que podem conduzir o residente a uma comunicação verbal e não verbal adequada para um relacionamento assertivo que permita atitudes críticas e reflexivas, além de ampliar a atuação gerencial dos recursos disponíveis para realização da assistência de enfermagem qualificada e segura^(16)^.

Outro estudo pontuou que as competências do enfermeiro neonatologista são organizadas em domínios que incluem a prática ética e legal, prática clínica, gestão, liderança, trabalho em equipe, pesquisa, produção de conhecimento, e prática educativa^(12)^. Os dados deste estudo identificam as competências, induzindo à inserção de metodologias que promovam um aprendizado relevante em torno dessas competências ao longo da sua especialização. Diante disso, uma das formas de garantir que essas competências sejam desenvolvidas na especialização é a introdução de um instrumento de avaliação como o ICEN. E por meio de análise comparativa, os domínios apresentados por este estudo estão em consonância com as competências estabelecidas e construídas no próprio ICEN, que também considera o nível de competência do residente de forma gradual.

Torna-se necessário conhecer os instrumentos e os métodos aplicáveis na avaliação da competência profissional em enfermagem. As metodologias de avaliação implicam a manutenção das ações e estratégias do ensino aprendizagem e modificam as propostas de qualificação profissional^(17)^.

Recentemente, a educação em enfermagem aborda uma educação baseada em competências. O currículo baseado em competências permite uma compreensão dos conhecimentos, habilidades e experiências, em que os alunos são avaliados criticamente pelas atribuições, o que proporciona o aprimoramento do pensamento crítico e oferece oportunidades de treinamento^(18,19)^.

Outro ponto baseia-se nas Diretrizes Gerais para os Programas de Residência Multiprofissional e em Profissional de Saúde, especificando que o projeto pedagógico de um programa de residência deve prever metodologias de integração de saberes e práticas que permitam construir competências compartilhadas, tendo em vista a necessidade de mudanças nos processos de formação, atenção e gestão na saúde^(20)^.

Foi observado que, na avaliação dos itens do ICEN, realizada pelos juízes, ocorreram algumas modificações nos itens, e outros itens do instrumento não sofreram alteração. Isso aconteceu porque houve reflexões dos especialistas acerca da avaliação adequada do enfermeiro e de como deve ser o instrumento de avaliação que possibilita uma aprendizagem e acompanhamento do ensino na residência, para que atenda ao referencial teórico e prático dos profissionais. As modificações existentes foram criadas a partir de uma única rodada entre os juízes, pois foi possível nessa rodada alinhar o conteúdo ao objetivo proposto.

Dessa forma, o instrumento de avaliação do estudo possui fundamentação, tornando-se válido considerando o seu conteúdo, já que contém aparência e clareza em sua estrutura, além de abrangência e pertinência dos conteúdos relevantes, que devem ser pontuados na residência, mantendo a capacidade de ser aplicável por programas de residência em enfermagem neonatal. O objetivo é auxiliar coordenadores, tutores e preceptores nos processos de avaliação do residente durante o cenário de prática, o que permite proceder para testar suas propriedades psicométricas.

### Limitação do estudo

Como limitação do estudo, destaca-se a ausência de instrumentos com o mesmo escopo que permitiriam a comparação dos resultados ou a realização de estudo comparativo. Outra limitação é que o ICEN prevê a avaliação de competências do residente enfermeiro, restringindo sua aplicação nos programas de residência uniprofissional de enfermagem em neonatologia.

### Contribuição para as áreas da enfermagem e saúde

O estudo apresenta implicações para pesquisas futuras, pois aborda temas relacionados à assistência do enfermeiro neonatologista, gestão e administração na área de neonatologia, e ensino na especialização, com foco na residência de enfermagem em neonatologia.

## CONCLUSÕES

Este estudo validou o conteúdo do ICEN após as análises e sugestões dos juízes especialistas. Desse modo, a configuração final do instrumento possui 26 objetivos específicos e 168 atividades e atitudes fundamentais para construção da aprendizagem do enfermeiro residente em neonatologia no período de 24 meses.

Considerou-se o ICEN como uma alternativa válida e confiável para avaliar o enfermeiro especialista. Espera-se que o ICEN possa ser aplicado para os residentes de enfermagem em neonatologia nos programas de residência com o intuito de ser um facilitador na avaliação das competências, favorecendo o ensino da enfermagem e auxiliando o residente no seu próprio desenvolvimento, onde o residente edifica sua própria reflexão e análise crítica do conhecimento transmitido.

## Data Availability

Os dados de pesquisa estão disponíveis em repositório: https://doi.org/10.48331/scielodata.6MRF78.
